# Gel-Based Purification and Biochemical Study of Laccase Isozymes from *Ganoderma* sp. and Its Role in Enhanced Cotton Callogenesis

**DOI:** 10.3389/fmicb.2017.00674

**Published:** 2017-04-20

**Authors:** Amit Kumar, Deepti Singh, Krishna K. Sharma, Sakshi Arora, Amarjeet K. Singh, Sarvajeet S. Gill, Barkha Singhal

**Affiliations:** ^1^Laboratory of Enzymology and Recombinant DNA Technology, Department of Microbiology, Maharshi Dayanand UniversityRohtak, India; ^2^School of Biotechnology, Gautam Buddha UniversityGreater Noida, India; ^3^Centre for Genetic Manipulation of Crop Plants, University of Delhi South CampusNew Delhi, India; ^4^Stress Physiology and Molecular Biology Laboratory, Centre for Biotechnology, Maharshi Dayanand UniversityRohtak, India

**Keywords:** *Ganoderma lucidum*, laccase isozymes, free radicals, molecular modeling and docking, callogenesis, cotton, MALDI-TOF MS

## Abstract

Basidiomycetous fungi, *Ganoderma lucidum* MDU-7 and *Ganoderma* sp. kk-02 secreted multiple laccase isozymes under diverse growth condition. Aromatic compounds and metal salts were also found to regulate the differential expression of laccase isozymes from both the *Ganoderma* sp. Laccase isozymes induced in the presence of copper from *G. lucidum* MDU-7 were purified by gel-based (native-PAGE) purification method. The purity of laccase isozymes was checked by zymogram and SDS-PAGE. The SDS-PAGE of purified proteins confirmed the multimeric nature of laccase isozymes. The molecular mass of isozymes was found to be in the range of 40–66 kDa. Further, the purified laccase isozymes and their peptides were confirmed with the help of MALDI-TOF peptide fingerprinting. The biochemical characterization of laccase isozymes viz. Glac L2, Glac L3, Glac L4, and Glac L5 have shown the optimum temperature in the range of 30°–45°C and pH 3.0. The *K*_m_ values of all the laccase isozymes determined for guaiacol were (96–281 μM), ABTS (15–83 μM) and *O*-tolidine (78–724 μM). Further, laccase isozymes from *G. lucidum* whole genome were studied using bioinformatics tools. The molecular modeling and docking of laccase isozymes with different substrates showed a significant binding affinity, which further validates our experimental results. Interestingly, copper induced laccase of 40 U/ml in culture medium was found to significantly induce cotton callogenesis. Interestingly, all the laccase isozymes were found to have an antioxidative role and therefore capable in free radicals scavenging during callogenesis. This is the first detailed study on the biochemical characterization of all the laccase isozymes purified by a gel-based novel method.

## Introduction

Laccases (EC 1.10.3.2, *p*-diphenol oxidase) are copper containing polyphenol oxidases that catalyze the oxidation of phenolic compounds with a concomitant reduction of molecular oxygen to water (Thurston, 1994). It is widely reported in filamentous fungi, yeast, plants, insects and bacteria; with diversified functions, i.e., depolymerization, polymerization, pathogenicity, melanin synthesis and spore coat formation (Thurston, 1994; Chung et al., 2008; Singh et al., 2014a). Wide industrial applicability of laccases in delignification, paper bleaching, deinking of newspaper, bioremediation, textile industries, biosensors, animal feed, and medical sectors approve this enzyme for basic research as well as novel biotechnological applications (Sharma et al., 2013; Singh et al., 2014b; Pezzella et al., 2015).

Laccases are extracellularly produced by white rot fungi in multiple isoforms. The isoforms are dependent on different culture conditions viz. temperature, pH, developmental stages and different phenolic and non-pheolic inducers (Dong et al., 2005; Piscitelli et al., 2011; Pezzella et al., 2013). Few attempts for purification and characterization of laccase isozymes have been made to understand their novel catalytic reactions (Ko et al., 2001; Mansur et al., 2003; Michniewicz et al., 2006; Ruhl et al., 2013; He et al., 2014; Kumar et al., 2015). Some protein purification techniques, i.e., ultra-filtration, ion-exchange chromatography, and hydrophobic interaction chromatography are very much in common use for purifications of laccase isozymes (Ramírez-Cavazos et al., 2014). Recently, Hernández-García and coworkers has reported the purification of lipase isoenzymes by adsorption on C8 magnetic particles, although some isoenzymes were lost during washing steps because of low hydrophobicity (Hernández-García et al., 2014). The laccase isozymes have moreover similar molecular weight and surface charges thus, it becomes more difficult to purify all the protein with conventional techniques. Therefore, the recent gel-based technique used in the current research work was found to be more efficient for the purification of all the laccase isozymes with narrow difference in charge and mass.

Previous studies suggest that the free radicals have an important role in plant tissue culturing, however, uncontrolled or in the absence of antioxidants, it may lead to the cellular dysfunction (Benson, 2000). Plants develops callus in the presence of certain biotic or abiotic stimulus (Ikeuchi et al., 2013). Cotton genotype, culture medium, growth regulator, ex-plant type, and carbohydrate type plays an important role in callogenesis (Michel et al., 2008). The Coker cotton lines are the most responsive for callogenesis and considered the initial step of somatic embryogenesis (Trolinder and Goodin, 1987).

In the present investigation, fermentation media supplemented with different aromatic compounds were used to induce and characterize laccase isozymes from *Ganoderma lucidum* MDU-7 and *Ganoderma* sp. kk-02. Furthermore, the methodology for gel-based i.e., native-PAGE purification of laccase isozymes has been developed. This is the first report on the purification, biochemical characterization, and molecular docking of all the fungal laccase isozymes from *G. lucidum* MDU-7 and their role in cotton callogenesis.

## Materials and methods

### Chemicals

Guaiacol, 2,2′-azino-bis(3-ethylbenzothiazoline-6-sulfonic acid) (ABTS), *O*-tolidine, D-quinic acid, acetylsalicylic acid, 3,5-Dihydroxytoluene, orcinol, 3,4-Dihydroxybenzoic acid, catechol, *O*-toluidine and tannic acid were purchased from sigma Co., (St. Louis, MO, USA). All other media components and chemicals were of highest purity grade available commercially.

### Microorganisms and culture conditions

Laccase producing basidiomycetous fungus, *G. lucidum* MDU-7 (Genbank accession no. KF549493) submitted to national fungal culture collection of India (NFCCI) (NFCCI accession no. 3873) was obtained from a culture collection of laboratory of Enzymology and Recombinant DNA Technology, Department of Microbiology, Maharshi Dayanand University, India. Whereas, *Ganoderma* sp. kk-02 (accession no. AJ749970) was obtained from a culture collection of Lignocellulose Biotechnology Laboratory, Department of Microbiology, University of Delhi, South Campus, India. Both of the fungal cultures, i.e., *G. lucidum* MDU-7 and *Ganoderma* sp. kk-02, were maintained on malt extract agar at 30°C, as described earlier (Kumar et al., 2015).

### Analytical procedure

Guaiacol was used as a substrate for assaying laccase activity following the method as described earlier (Sharma et al., 2005). One unit (U) of laccase was defined as the change in absorbance of 0.01 ml^−1^ min^−1^ at 470 nm.

### Zymogram of laccase isozymes

Activity staining for laccase was performed by staining the native-PAGE gel with 0.1 M citrate-phosphate buffer (pH 4.0) containing 2 mM *O*-tolidine and incubated at 30°C in dark conditions (Kumar et al., 2015).

### Effect of fermentation volume on production time

Laccase production was carried out in 50 and 25 ml malt extract broth (MEB) in 250 ml Erlenmeyer flask in static culture conditions. Flask with 50 and 25 ml of MEB were inoculated with eight and four fungal discs (8 mm each), respectively, from the periphery of 5 days old culture of *G. lucidum* MDU-7 and *Ganoderma* sp. kk-02.

### Effect of pH on laccase isozymes production

Production of laccase isozymes was studied from both *Ganoderma* sp. at different pH (2.5–5.0) in static culture conditions. Crude enzyme samples were harvested from the 2nd day onwards up to the 14th day for zymogram analysis of laccase isozymes.

### Effect of temperature on laccase isozymes production

Production of laccase isozymes was studied from both *Ganoderma* sp. at different incubation temperature (25°–35°C) in static culture conditions. Crude enzyme samples were harvested from the 2nd day onwards and up to the 14th day for zymogram profile analysis of laccase isozymes.

### Effect of different phenolics and non-phenolics compounds on laccase isozymes production

The culture medium was induced on the 3rd day with several aromatic compounds (1 mM), CuSO_4_ (7 mM), and ethanol (3%). Crude enzyme samples were harvested from the 4th day onwards up to the 14th day for zymogram analysis of laccase isozymes.

### Gel-based purification of laccase isozymes produced

**Figure 1:**
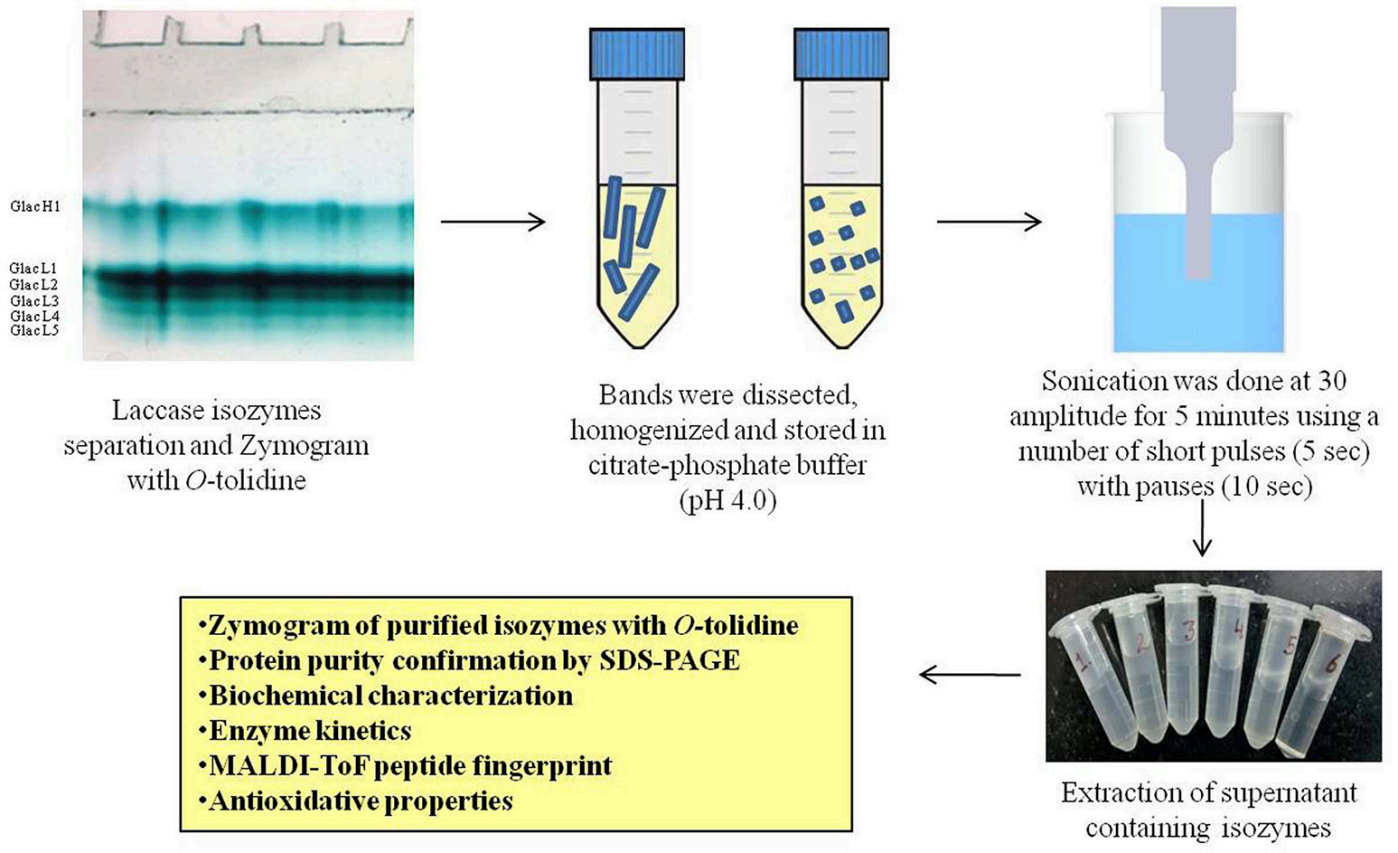
**Schematic representation for purification of laccase isozymes**.

The copper induced culture broth was filtered through Whatman filter no. 1 and centrifuged at 13,000 × g for 15 min at 10°C. The protein extract was concentrated using an Amicon Ultra-15 membrane filter (Millipore, Germany). Partial purification of laccase from the culture filtrate was carried out by the addition of finely ground ammonium sulfate at three different saturation levels, i.e., 0–20%, 20–40%, and 40–80%. After overnight incubation at 4°C, the fraction 40–80% containing high laccase activity was centrifuged at 9,000 × g for 20 min. Precipitates were dissolved in 20 mM citrate-phosphate buffer (pH 4.0) and dialyzed overnight against the same buffer at 4°C. Further, laccase isozymes were purified from the modified method of native-PAGE (12%) purification as reported elsewhere (Kumar et al., 2015; Figure 1). After native-PAGE, the gel was stained with 0.1 M citrate-phosphate buffer (pH 4.0) containing 2 mM *O*-tolidine and incubated at 30°C in dark conditions (Kumar et al., 2015). SDS-polyarylamide gel electrophoresis (SDS-PAGE) was used to determine the purity and molecular mass of laccase isozymes. Further, SDS-PAGE was performed for purified laccase isozymes under both unreducing and reducing conditions to confirm the multimeric nature. In SDS-PAGE, the protein bands were visualized with Coomassie Brilliant Blue R-250 colloidal staining and destained with a solution containing 25% methanol as described elsewhere (Neuhoff et al., 1988).

### UV absorption spectra of purified laccase isozymes

The laccase UV-absorbance spectrum was scanned from 300 to 700 nm at room temperature on a Shimadzu UV-1800 spectrophotometer.

### Identification of laccase isozymes by MALDI-TOF analysis

The identification of proteins by peptide mass fingerprinting (MALDI-TOF/TOF analysis) of purified and partially purified laccase isozymes expressed during co-culture conditions. The desired bands obtained by SDS-PAGE analysis were cut and trypsin digested. The resulted samples were spotted on a MALDI target plate to obtain peptide spectra using ABI SCIEX MALDI-TOF/TOF 5800. The peptide sequences were then analyzed by comparing mass spectrometry data from the National Center for Biotechnology Information (NCBInr) protein database using the Mascot search algorithm (Cottrell and London, 1999).

### Biochemical characterization of purified laccase isozyme

The enzyme activity was assayed in the pH range of 3.0–10.0 using suitable buffers. The substrate was prepared in 50 mM of citrate-phosphate buffer (pH 3.0–6.0), phosphate buffer (pH 7.0), Tris-HCl (pH 8.0–9.0) and glycine-sodium hydroxide buffer (pH 10.0). Effect of temperature on isozyme activity were determined by incubating the reaction mixture at different temperature varying from 25° to 60°C under standard assay conditions.

The pH stability was determined by incubating the enzyme in buffers of 50 mM of citrate phosphate buffer (pH 3.0–6.0), phosphate buffer (pH 7.0), Tris-HCl (pH 8.0–9.0) and glycine-sodium hydroxide buffer (pH 10.0) 30 min at 30°C. Whereas, the temperature stability was determined by incubating the enzyme samples at various temperatures (25°–60°C) for different time intervals (1–8 h). The enzyme activity was expressed as percent relative activity with respect to maximum activity, which was considered as 100%.

Michaelis-Menten constant (*K*_m_) and the maximum rate of reaction (V_max_) were determined by using guaiacol (470 nm) and *O*-tolidine (627 nm) as a substrate, at concentration ranging from 0.5 to 3.0 mM and 0.1–0.8 mM, respectively, in citrate-phosphate buffer of pH 4.0. Whereas, *K*_m_ and V_max_ were determined by ABTS (420 nm) as a substrate at concentration ranging from 0.02 to 0.5 mM in 0.1 M sodium acetate buffer of pH 5.0. The values of *K*_m_ and V_max_ were calculated from the Eadie-Hofstee plot.

### Laccase isozymes sequence analysis

The protein sequences of *G. lucidum* laccase isozymes were retrieved from http://www.herbalgenomics.org/galu (Chen et al., 2012). The physiochemical properties of different laccase isozymes were computed using the expasy protparam server at http://web.expasy.org/protparam (Gasteiger et al., 2005).

### Homology modeling and molecular docking of laccase isozymes

The three dimensional structure of laccase isozymes were generated with software Modeller ver. 9.14 using different templates, obtained from PDB database, based on their homology with the target sequences. The models generated were analyzed for quality check using ProSa (Wiederstein and Sippl, 2007), ProQ (Wallner and Elofsson, 2003) and RAMPAGE (Abagyan et al., 1994) online servers. Molecular docking of the best models was done with laccase substrates, i.e., ABTS, *O*-tolidine and guaiacol using molsoft ICM (internal coordinate mechanics). The flexible docking was done by optimizing a set of ligand internal coordinates in the space of grid potential maps calculated for the protein pockets using Monte Carlo simulations (Abagyan and Totrov, 1994; Abagyan et al., 1994; An et al., 2005).

### Effect of fungal laccase in cotton callogenesis

#### Plant material

Cotton seeds (*Gossypium hirsutum* L.) Coker 310FR line (Kumar et al., 1998) was delinted with sulfuric acid and washed thoroughly with tap water for 30 min. Delinted seeds were surface sterilized by dipping in sterile distilled water with a few drops of Tween 20 for 10 min. followed by 5 to 7 rinses in sterile distilled water. They were subsequently treated with 70% ethanol for 30 s, followed by 0.1% mercuric chloride solution for 3 min. and then rinsed 7 times with sterile distilled water. The sterilized seed without seed coats was placed on 1/2 MS (Murashige and Skoog medium) medium at 28°C in dark for 2 days and transferred to culture room under 28° ± 2°C; 750 lux light intensity and photoperiod of 16 h light/8 h dark for 7 days.

#### Callus initiation

Cotyledonary explants of seven day old plants were excised and subjected for callogenesis following the protocol and media described by Chaudhary et al. (2003). Different units of filter sterilized Cu^2+^ induced partially purified fungal laccase were added in the MST1 media before pouring on petri plates. Multiple (15) biological replicates were taken for callogenesis. Callus was initiated on MST1 medium and was grown at 28° ± 2°C, 1000 lux light intensity and 16-h light/8-h dark photoperiod for 30 days.

### Antioxidant property of laccase isozymes

BSA (50 μg) was added in 0.1 ml of 0.1 M citrate-phosphate buffer, pH 5.20 containing 100 μM copper and 2.5 mM hydrogen peroxide. Purified laccase isozymes (5 unit) were added and incubated for 2 h at 37°C. Ascorbate (50 μg/0.1 ml) were used as a control in the absence and presence of laccase isozymes. BSA (2 μg) from each reaction mixture was run on 12% SDS-PAGE gel (Bio-rad mini, USA) along with markers (Sigma-Aldrich, USA), and were visualized by staining with CBBR-250.

### Statistical analysis

The average weights of callus mass have been shown as mean values ± standard deviation (SD). Data collected in the experiments were statistically analyzed using one-way ANOVA test. The means obtained for different events were compared using a paired *t*-test. All analyses were performed by using Microcal Origin Version 6.0. *A*-value of *P* < 0.05 was considered to be statistically significant.

## Results and discussion

### Effect of fermentation volume on time period of production of laccase

**Figure 2:**
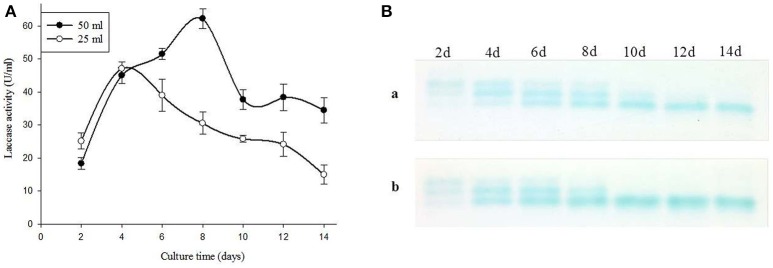
**(A)** Effect of fermentation volume on laccase production and **(B)** isozymes secretions (a. 50 and b. 25).

*Ganoderma lucidum* MDU-7 was grown in 50 ml and 25 ml of fermentation media in static conditions to study the effect of fermentation volume on the maximum day of laccase production. Interestingly, the time period of maximum laccase production decreases to half when media volume was reduced to 25 ml (47 U/ml) from 50 ml (62 U/ml) (Figure 2). Similarly, *Ganoderma* sp. kk-02 also showed the reduction in laccase production time from the 12th day (32 U/ml) to 6th day (17 U/ml) (data not shown). Furthermore, the zymogram showed the similar isozymes patterns in both 50 and 25 ml culture media (Figure 2B). Earlier studies have shown that the white rot fungus takes more time to reach the peak of laccase production (Sharma et al., 2013). The reduction in production time for laccase from *Ganoderma lucidum* MDU-7 and *Ganoderma* sp. kk-02 has positive implications in the study of isozymes enzyme properties and its biotechnological applications.

### Effect of pH on production of laccase isozymes

**Figure 3:**
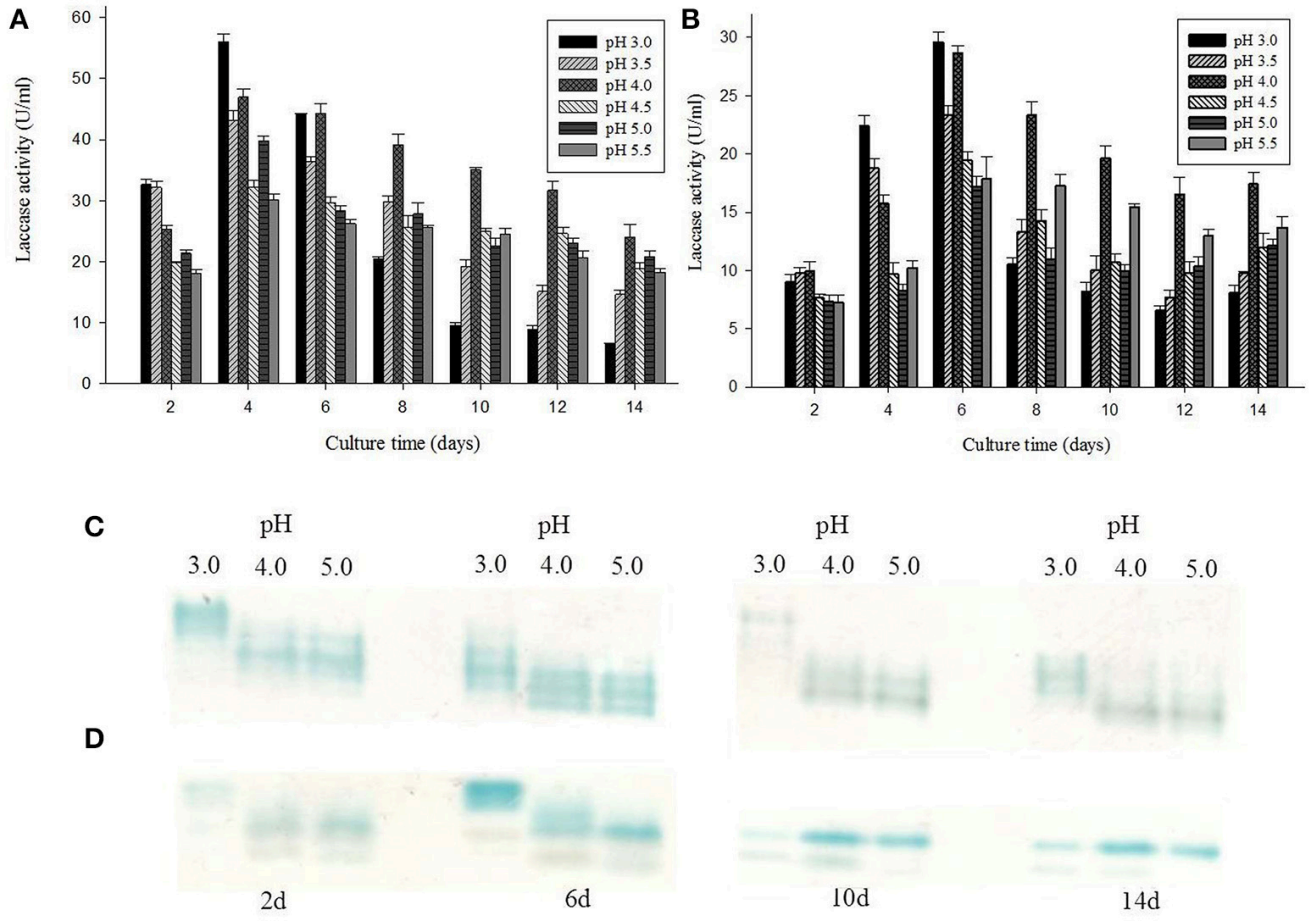
**Effect of different pH conditions on laccase production and isozymes secretion from (A,C)** *G. lucidum* MDU-7 and **(B,D)** *Ganoderma* sp. kk-02.

Laccase isozymes from *G. lucidum* MDU-7 showed a differential band pattern on the native gel, when grown in different pH conditions, with maximum laccase activity (56 U/ml) at pH 3.0 (Figure 3A). A comparative study of laccase isozymes, harvested at different time intervals showed a unique isozyme pattern at pH 3.0, while isozyme secretion pattern was somewhat similar in all other pH conditions (Figure 3C). Similarly, *Ganoderma* sp. kk-02 also revealed a unique isozyme pattern of laccase at pH 3.0 (30 U/ml) and follows the uniform pattern of laccase isozymes at all the study pH (Figures 3B,D). The mycelial growth and laccase activity of both *Ganoderma* sp. were optimum at pH 4.0 and therefore selected for further isozymes studies. Recently, Sharma and co-workers have reported differential regulation of laccase isozymes from *G. lucidum* MDU-7 at pH 5.2 in time-dependent manner (Kumar et al., 2015). In general, the production of laccase in basidiomycetous fungi is favored at acidic pH (Sharma et al., 2013; Kumar et al., 2015), but the secretion of isozymes pattern was under the regulation of broad pH, which indicate the specific function of these isozymes.

### Effect of temperature on production of laccase isozymes

**Figure 4:**
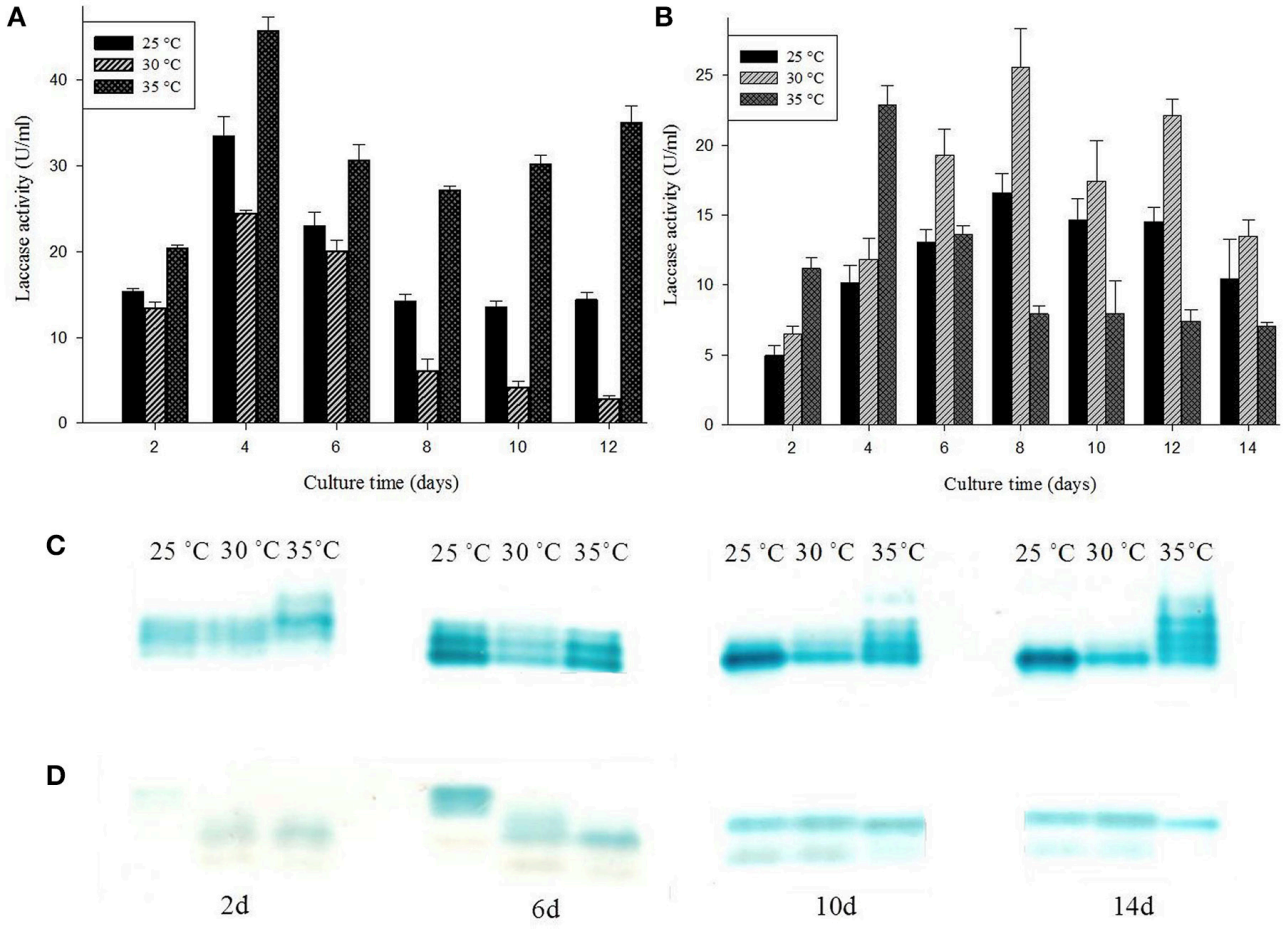
**Effect of different temperature conditions on laccase production and isozymes secretion from (A,C)** *G. lucidum* MDU-7 and **(B,D)** *Ganoderma* sp. kk-02.

*G. lucidum* MDU-7 has similar laccase isozyme secretion patterns at 25° and 30°C, but it has also been found to secrete additional isoforms of laccase at 35°C. Comparative study at 35°C has shown somewhat higher mol mass laccase isozymes secreted initially; further these isozymes re-appeared on later stages during media starving conditions (Figures 4A,C). Contrary to this *Ganoderma* sp. kk-02 produces different isoforms of laccase on the initial days at 25°C (Figures 4B,D). Higher secondary metabolic product, and restricted mycelium growth were observed for both *Ganoderma* sp. at 25° and 35°C. Therefore, 30°C was selected as the optimum temperature for further isozyme studies. Temperature is an important environmental factor which regulates the secretion of laccase isozymes (Fonseca et al., 2013). Earlier, optimum temperatures for maximum laccase production have been reported to be species specific i.e., 30°C for *P. ostreatus, T. modesta, Cyathus bulleri, Phlebia brevispora, Ganoderma* sp. kk-02 (Nyanhongo et al., 2002; Sharma et al., 2005; Vasdev et al., 2005; Šnajdr and Baldrian, 2007) and 35°C for *T. versicolor* (Šnajdr and Baldrian, 2007). But, the reports on the effect of temperature on individual laccase isozyme secretion are very few. Recently, Fonseca et al. (2013) have reported the effect of different temperature on the secretion of laccase isozymes from several white rot fungi viz. *C. versicolor, P. brevispora, G. applanatum*, and *Pycnoporus sanguineus*.

### Effect of aromatic compounds on production of laccase isozymes

**Figure 5:**
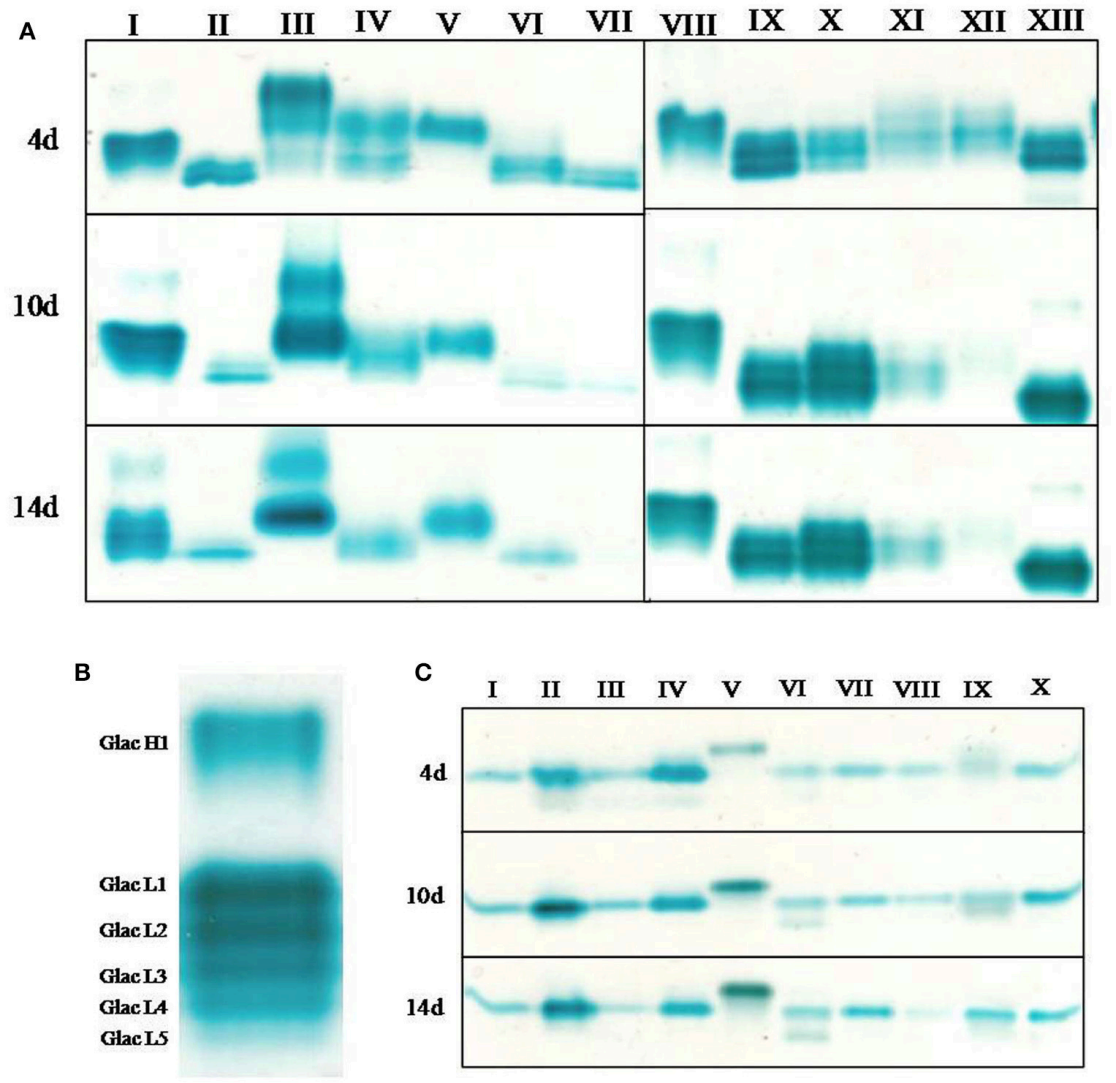
**The comparative study on effect of different aromatic compounds on laccase isozymes secretion. (A)** *G. lucidum* MDU-7. I, CuSO_4_; II, control (pH 4.0); III, tannic acid; IV, 3,5-dihydroxytoluene; V, 3,4-dihydroxybenzoic acid; VI, orcinol; VII, quinic acid; VIII, CuSO_4_; IX, acetylsalicylic acid (1 mM); X, acetylsalicylic acid (5 mM); XI, catechol; XII, ethanol (3%); XIII, *O*-toluidine. **(B)** *G. lucidum* MDU-7 induced with 7 mM CuSO_4_ (adopted from Kumar et al., 2015). **(C)** *Ganoderma* sp. kk-02. I, control (pH 4.0); II, CuSO_4_; III, quinic acid; IV, *O*-toluidine; V, tannic acid; VI, catechol; VII, 3,5-dihydroxytoluene; VIII, ethanol (3%); IX, 3,4-dihydroxybenzoic acid; X, orcinol.

*Ganoderma lucidum* MDU-7 was reported to produce six laccase isozymes in the presence of Cu^2+^ (Kumar et al., 2015; Figure 5B) and therefore was further used as a reference for identification of a novel isozymes in the presence of several aromatic compounds. Laccase isozymes under the presence of different inducible compounds have been summarized in Table 1. Tannic acid was found to be important for induction of novel higher mol mass laccase isozymes in both *Ganoderma* sp., especially in *Ganoderma* sp. kk-02 (Figure 5). In *G. lucidum* MDU-7, *O*-toluidine was found to induce a novel laccase isozyme i.e., Glac L6, on the 4th day, which was not reported to be induced by other phenolic compounds used in the present study. Whereas, another strain of *Ganoderma* i.e., *Ganoderma* sp. kk-02, did not produce any additional laccase isozyme in the presence of *O*-toluidine. In the current study, aromatic compounds were found to be responsible for differential expression of laccase isozymes in species specific and time-dependent manner (Figure 5). Aromatic and phenolic compounds structurally related to lignin monomers, long far have been used for the production of laccase enzyme (Piscitelli et al., 2011). But there are very few reports which focus on the induction of laccase isozymes by using various aromatic compounds and metal salts (Sharma et al., 2005; Piscitelli et al., 2011; Kumar et al., 2015). Earlier reports revealed that certain aromatic compounds i.e., vanillin, ferulic acid (De Souza et al., 2004) and 2,5-xylidine, produces several laccase isozymes from selected basidiomycetous fungi (Yaver et al., 1996). However, Xiao et al. (2004) reported the selective induction of lac A by *O*-toluidine and lac B by 3,5-dihydroxytoluene. Tannic acid, present in the bark of trees plays an important role against pathogenic infection (Chung et al., 2008). Earlier, pathogenic fungus *Cryphonectria parasitica* has been reported to induce *lac*3 gene only in the presence of tannic acid, which probably has an important role to play in fungal virulence of the chestnut blight (Chung et al., 2008). Therefore, it can be hypothesized that the species-specific action of different aromatic compounds in the production of laccase isozymes might be due to differences in ecological habitat, which eventually helps in the adaptation of the fungus. Despite the well-known role of laccase in lignin degradation, our observations and reports suggest more specific roles of laccase isozymes (Kumar et al., 2015). The actual mechanism and purposes of time dependent and substrate specific laccase isozyme secretion is unknown and therefore needs to be scientifically elaborated and ecologically related.

**Table 1:** **Effect of inducers on ***G. lucidum*** MDU-7 for the production of high molecular mass (Glac H) and low molecular mass (Glac L) laccase isozymes**.

**Inducers**	**Concentration (mM)**	**Time (days)**	**Laccase isozymes (Glac)**
Control	0	4	L4, L5
		10	L5
		14	L5
Tannic acid	1.0	4,10,14	H2, L1, L2
3,5-dihydroxytoluene	1.0	4	H2, L3, L4
		10,14	L3, L4, L5
3,4-dihydroxybenzoic acid	1.0	4	H2
		10, 14	L1
Orcinol	1.0	4, 10	L4, L5
		14	L5
Quinic acid	1.0	4	L4, L5
		10, 14	L5
Acetylsalicylic acid	1.0	4,10	L3, L4, L5
		14	H2, L3, L4, L5
Catechol	1.0	4	L1, L2, L3
		10, 14	L4, L5
Ethanol	3%	4	L1, L2, L3, L4
		10, 14	L1, L2
*O*-toluidine	1.0	4	L4, L5, L6
		10, 14	H2, L4, L5
CuSO^*^_4_	7.0	4	L3, L4, L5
		10, 14	H1, L1, L2, L3, L4, L5

* *Adopted from Kumar et al. (2015)*.

### Gel-based purification of laccase isozymes and their identification

Among different phenolics and non-phenolics compound studied in the present investigation, copper has shown a significant effect on the high laccase isozymes production from *G. lucidum* MDU-7 (Kumar et al., 2015). Furthermore, six laccase isozymes, i.e., Glac H1, Glac L1, Glac L3, Glac L4, and Glac L5 were purified from gel-based (native-PAGE) method (Figure 1). The UV spectrum studies of the laccase isozymes showed a broad peak around 330 nm regions due to the presence of type 3 copper atom (Supplementary Figure 1; Solomon et al., 2014).

**Figure 6:**
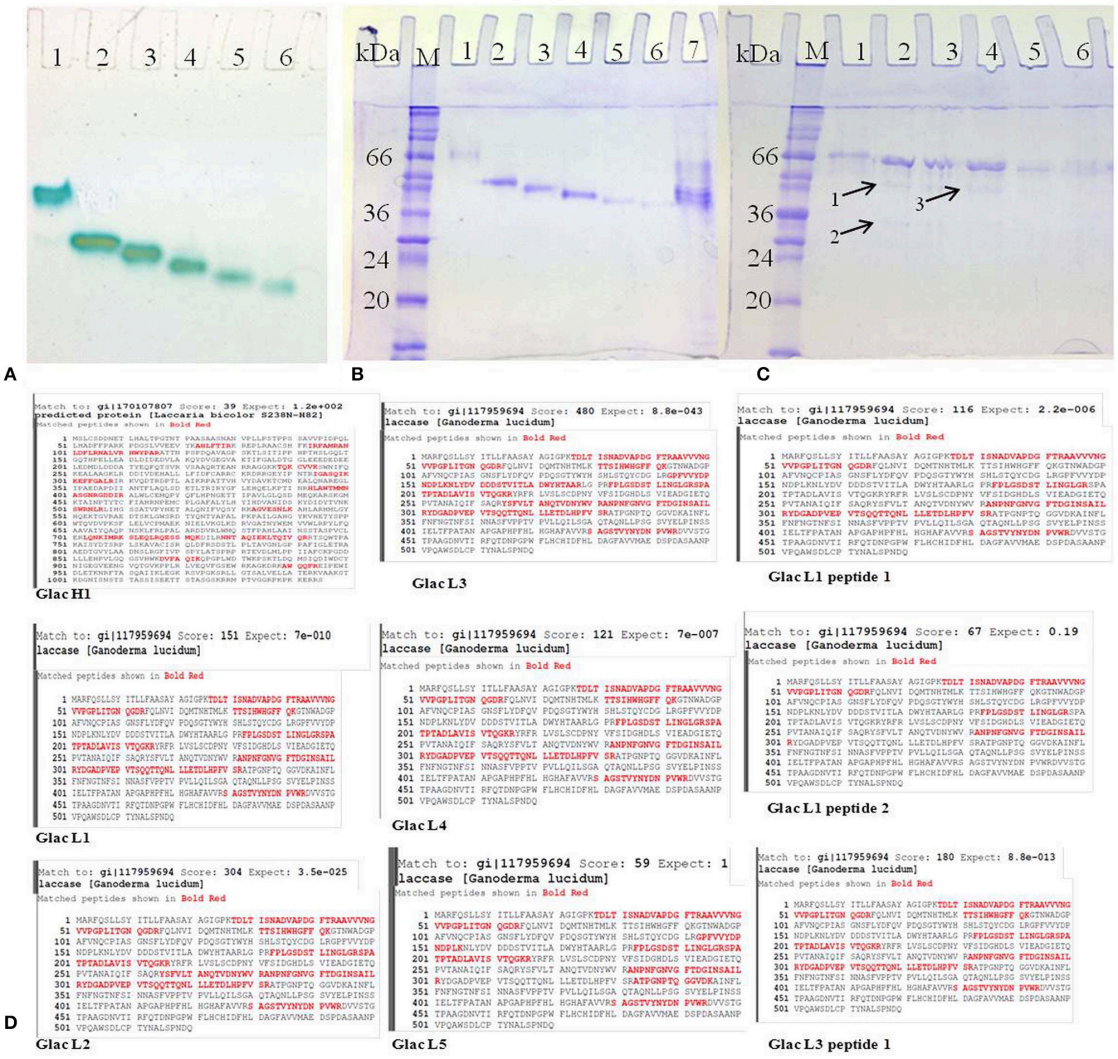
**Purified laccase isozymes (A)** 1-6, activity staining with *O*-tolidine **(B)** 1-7, SDS PAGE in unreduced and non-denaturing conditions **(C)** 1-6, SDS PAGE in reduced conditions; A1,B1,C1, Glac H1; A2,B2,C2, Glac L1; A3,B3,C3, Glac L2; A4,B4,C4, Glac L3; A5,B5,C5, Glac L4; A6,B6,C6, Glac L5; B7, crude partially purified protein. Arrow 1, Glac L1 peptide 1; Arrow 2, Glac L1 peptide 2; Arrow 3, Glac L3 peptide 1. (M, marker; kDa, kilo Dalton). **(D)** Confirmation of laccase isozymes by MALDI-TOF peptide finger printing.

All purified laccase isozymes showed a single band in native gel electrophoresis followed by activity staining (Figure 6A). The SDS-PAGE analysis of purified laccase isozymes has also revealed a single band after coomassie staining (Figures 6B,C). However, in the presence of reducing agent i.e., 2-mercaptoethanol, revealed the presence of more than one peptide in purified laccase isozymes (Figure 6C). All the purified laccase isozymes (Figure 6B) and peptides (Figure 6C) were identified by MALDI-TOF and the peptide fingerprints were compared from NCBI protein database (Figure 6C). The peptide sequence of purified proteins matched with laccase of *G. lucidum* (Figure 6D). Further, the laccase isozyme Glac H1 was found to be novel as the score (39) was not very high. The peptide sequencing also complement the earlier report on purified laccase isozymes (Glac H1 and Glac L1) from *G. lucidum* MDU-7 by using native-PAGE purification method (Kumar et al., 2015).

### Biochemical characterization and the kinetic parameters of purified laccase isozymes

**Figure 7:**
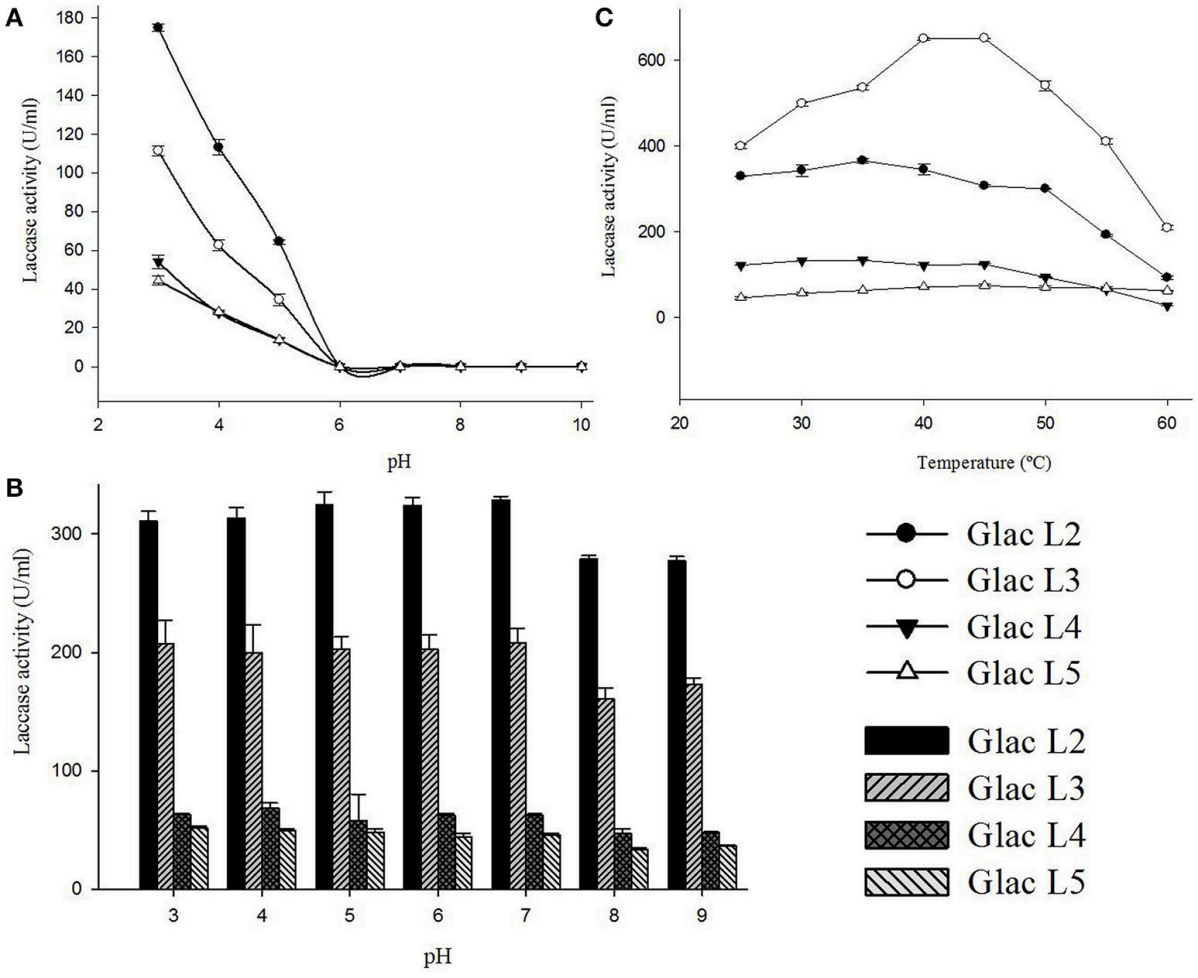
**Biochemical characterizations of laccase isozymes (Glac L2-Glac L5) from ***G. lucidum*** MDU-7. (A)** pH optima of laccase isozymes. **(B)** The pH stability of laccase isozyme. **(C)** Optimal temperature for laccase isozyme.

Purified laccase isozymes (Glac L2-Glac L5) were active at acidic pH, with optimum activity at pH 3.0 (Figure 7A). In the present study, laccase isozymes (Glac L2-Glac L5) were found to be stable over a wide pH range, i.e., 3.0–9.0 (Figure 7B). The laccase isozymes Glac L2 (35°C), Glac L3 (40°–45°C), Glac L4 (30°–35°C) and Glac L5 (40°–45°C), showed a wide range of temperature optima (Figure 7C).

The laccase isozyme, Glac L2-Glac L4, have pH optima similar to the earlier reports for Glac H1 (pH 4.0) and Glac L1 (pH 4.0) from *G. lucidum* MDU-7 (Kumar et al., 2015). Also, the isozymes showed broad pH stability. An earlier study on the effect of redox potential and hydroxide ion on the pH profile activity of fungal laccases revealed that at higher pH for oxidation of phenolic substrates, there is a difference in electrode reduction potential in T1 Cu and reducing substrates, whereas, OH^−^ inhibits the T2/T3 Cu center (Xu, 1997).

**Figure 8:**
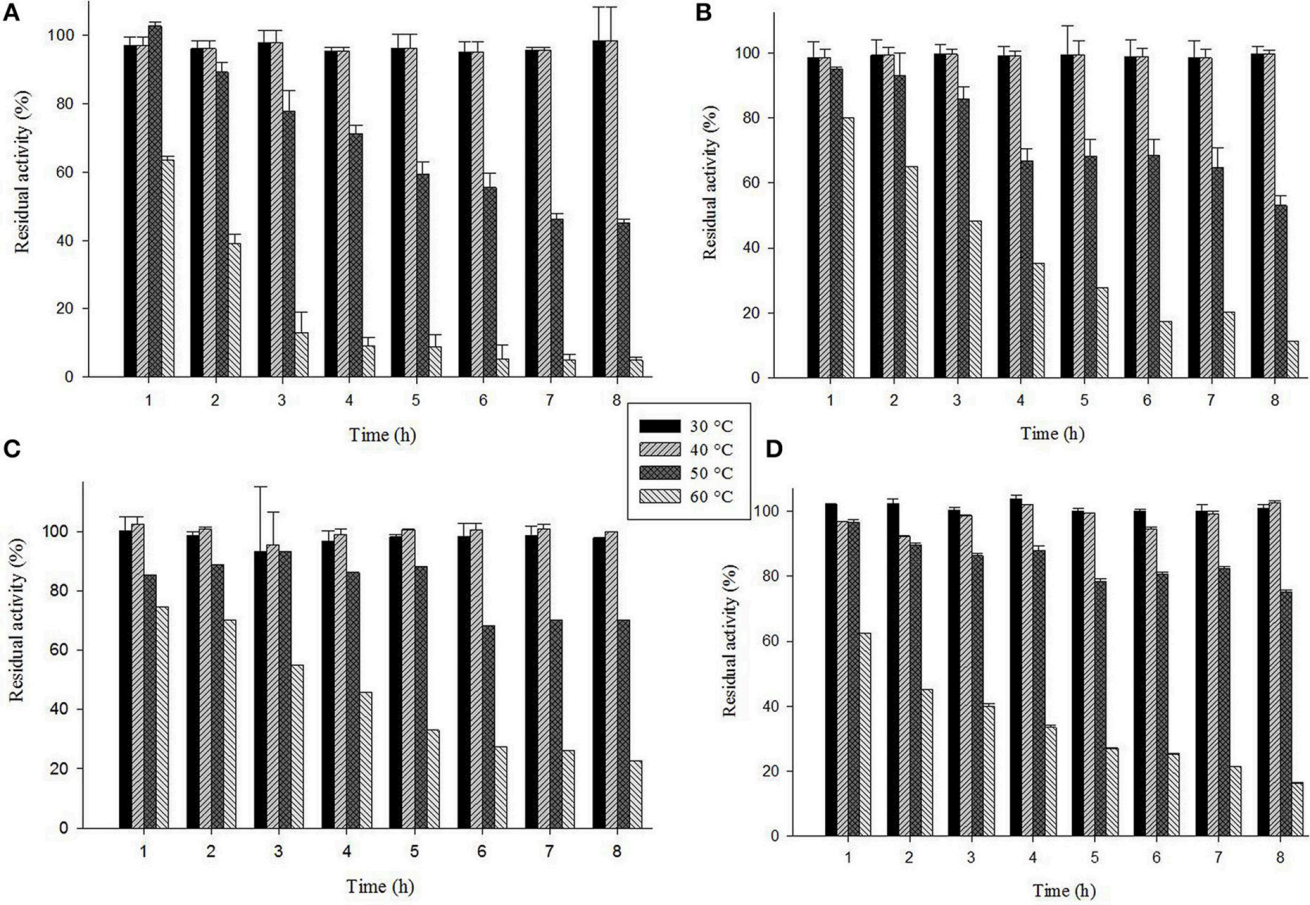
**Thermostability of laccase isozymes from ***G. lucidum*** MDU-7. (A)**, Glac L2; **(B)**, Glac L3; **(C)**, Glac L4; **(D)**, Glac L5.

The laccase isozyme showed different degree of temperature stability i.e., Glac L2 lost 50% after 6 h, while Glac L3, Glac L4, and Glac L5 had lost 30% activity after 8 h at 50°C, respectively, whereas, Glac L3, and Glac L4 lost 50% of residual activity after 3–4 h at 60°C (Figure 8). The kinetic study of purified laccase isozyme from *G. lucidum MDU-7* shows differential *K*_m_ and V_max_ values (Table 2). All the purified laccase isozymes revealed different catalytic properties, depending upon the atomic interaction with the substrate. Laccase isozymes, i.e., Glac L2 and Glac L5 have a high substrate affinity toward ABTS (83 μM and 15 μM), whereas, Glac L3 and Glac L4 (80 and 96 μM) have a high affinity toward guaiacol (Supplementary Figures 2–4). The results were also compared with the earlier enzyme kinetic reports on Glac H1 and Glac L1 from *G. lucidum* MDU-7 (Kumar et al., 2015).

**Table 2:** **Michaelis-Menten kinetic constants of studied laccase isozymes from ***G. lucidum*** MDU-7 at their optimal pH with different substrates**.

**Laccase Isozymes**	**ABTS**		***O*****-tolidine**		**Guaiacol**	
	***K*_m_ (μM)**	**V_max_ (μM/ml/min)**	***K*_m_ (μM)**	**V_max_ (OD/min/unit enzyme**	***K*_m_ (μM)**	**V_max_ (μM/ml/min)**
Glac H1^*^	29	0.152	338	0.44	281	18 × 10^4^
r^2^	0.974		0.946		0.970	
Glac L1^*^	26	0.780	320	1.7	98	32 × 10^4^
r^2^	0.993		0.985		0.985	
Glac L2	83	1.74	724	0.092	115	207 × 10^3^
r^2^	0.981		0.921		0.987	
Glac L3	81	1.7	507	0.0608	80	12 × 10^4^
r^2^	0.911		0.949		0.979	
Glac L4	50	0.72	365	0.023	96	365 × 10^3^
r^2^	0.924		0.940		0.949	
Glac L5	15	0.399	78	0.021	126	43 × 10^3^
r^2^	0.977		0.986		0.932	

* *Adopted from Kumar et al. (2015)*.

### Laccase isozymes sequence analysis

The fungal genome sequencing is considered to be a milestone in the field of basic biology and biotechnology (Sharma, 2015). Earlier, the whole genome of *G. lucidum* has been reported with a complete set of 36 ligninolytic oxidoreductases, among which 14 are the candidate gene for laccases (Chen et al., 2012). The analysis of physicochemical properties of different laccase isozymes showed a molecular mass ranging from 53.3 to 66.4 kDa and a theoretical pI of 4.5 to 5.38 (Table 3). The negatively charged residues were found to be more as compared to the positively charged residues in all the laccase isozymes. Also, the instability index of the isozymes was found to be below 40 and aliphatic index ranging 79.09–88.30 (Table 3). However, the isozymes, GL17426 had an instability index of 39.79. The *in silico* studies of laccase isozymes from *G. lucidum* was found to be similar to earlier experimental reports (Kumar et al., 2015). The instability index and aliphatic index values predicting the proteins to be highly stable even at higher temperatures, and thus suitable for different industrial applications. However, GL17426 instability index was very low, thus suggesting it to be not very stable. Previous reports on laccase and its isozymes also suggest to be stable at 60°C (Sharma et al., 2013; Kumar et al., 2015), although laccase isozyme Glac H1, Glac L2 have lower thermostability than Glac L1 (Kumar et al., 2015) Glac L3, Glac L4, and Glac L5 thus validates the above instability and aliphatic index values of laccase isozymes.

**Table 3:** **Physicochemical analysis of laccase isozymes**.

**Isozyme**	**Amino acids**	**Mol mass (kDa)**	**pI**	**+ve charged residues**	**−ve charged residues**	**Instability Index**	**Aliphatic Index**	**GRAVY**
GL16398	520	56.62	4.62	21	49	31.55	80.29	−0.125
GL29486	520	56.20	5.58	25	40	27.05	80.88	−0.081
GL29253	521	55.25	4.50	17	46	26.61	85.18	−0.096
GL21497	544	58.97	5.20	24	45	35.93	84.63	−0.025
GL29490	509	54.41	4.68	22	50	35.29	83.73	−0.021
GL30788	559	60.47	4.53	26	60	33.53	83.09	−0.042
GL29234	541	57.69	4.95	29	54	26.40	83.88	−0.050
GL23477	519	56.94	5.15	23	43	38.42	88.30	−0.044
GL17426	498	54.47	5.26	28	46	39.79	84.66	−0.025
GL18428	496	53.30	4.84	26	48	29.20	79.09	−0.085
GL16401	528	57.24	5.07	29	53	35.01	86.84	−0.138
GL22987	610	66.45	5.38	40	60	32.64	84.08	−0.209

Further, analysis of the laccase sequences revealed that all the isozymes possess several varied numbers of putative N-linked glycosylation sites, with the consensus sequence N-V-[TI] found in all of them (Sharma et al., 2016). In addition, several potential O-linked glycosylation sites were also identified with lowest sites in isozyme GL16401. Furthermore, analysis of other post-translational modifications showed diverse serine, threonine and tyrosine phosphorylation sites in different laccase isozymes (Sharma et al., 2016). Analysis of the laccase sequences revealed that all the isozymes have a varied number of putative N-linked glycosylation sites. The potential role of glycosylation has been reported in protein stability, activity and proper folding (Ceriotti et al., 1998; Kang et al., 2008). However, a recent report suggested that the glycan moieties play a significant role in catalytic efficiency, thermostability and enzyme activity (Vite-Vallejo et al., 2009; Maestre-Reyna et al., 2015). Post-translational modifications showed several potential serine, threonine and tyrosine phosphorylation sites in different isozymes. Phosphorylation of fungal laccase is linked to signaling cascades, protein-protein interactions, protein localization and regulation of gene expression (Hunter, 2000). The remarkable difference in the post-translational modifications in isozyme GL16401 could be due to the lowest percentage of threonine, which could eventually make the isozyme more conserved evolutionary and less prone to post-translational modifications.

### Homology modeling and molecular docking of laccase isozymes

**Figure 9:**
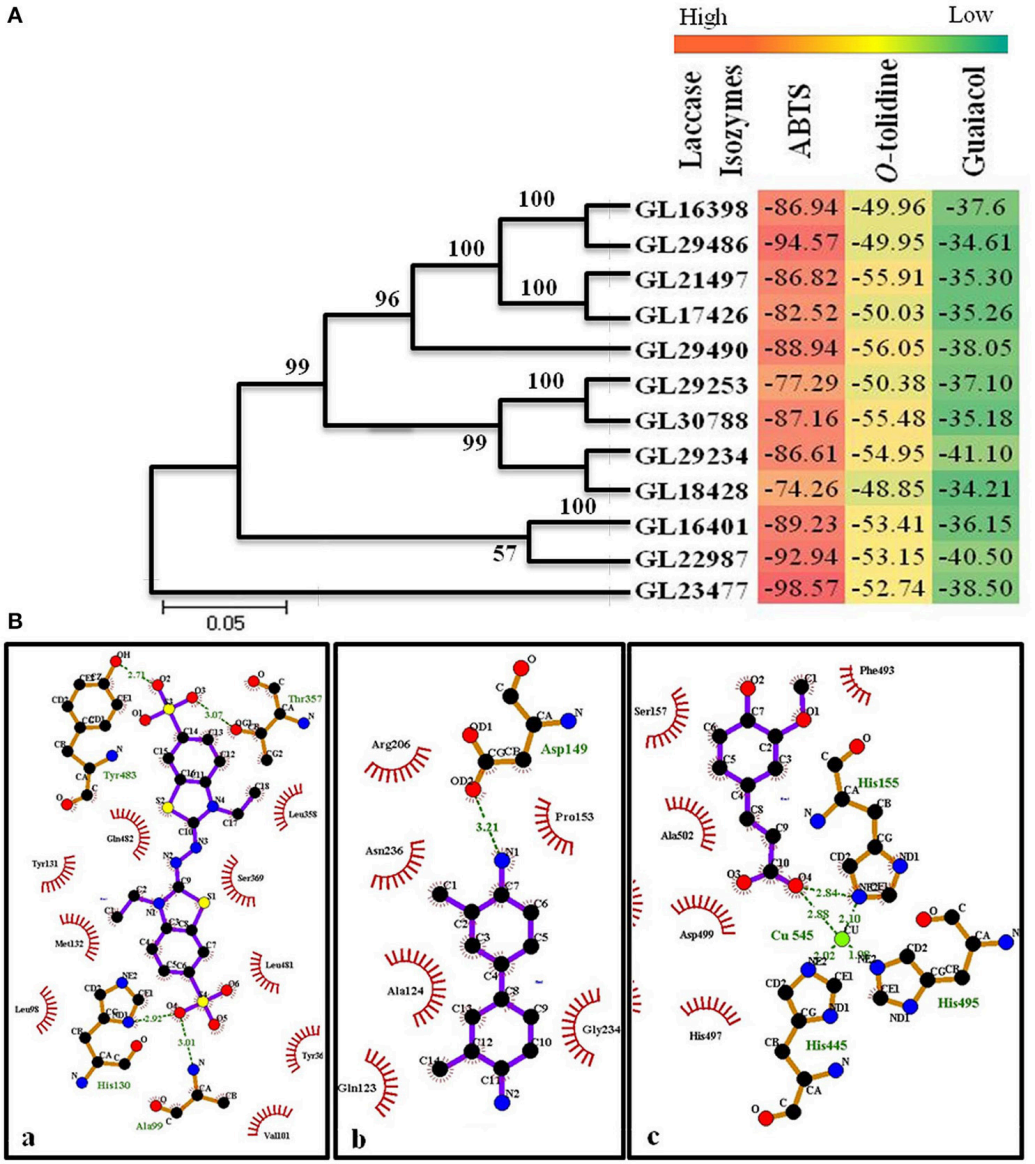
**(A)** Phylogenetic study and heat map representing molecular docking score of laccase isozymes with different substrates. **(B)** Molecular interaction of laccase isozymes with different substrates. Interaction of (a) isozyme GL29486 with substrate ABTS; (b) isozyme GL29490 with substrate *O*-tolidine; (c) isozyme GL29234 with substrate guaiacol. The green lines shows the hydrogen bonds between ligand and amino acids. The spoked arcs represent residues making non-bonded contacts with the ligand. (Ligplot^+^)

The homology modeling of the laccase isozymes was done and the models were initially selected based on their lowest molPdf value and DOPE (Discrete Optimized Protein Energy) scores (Supplementary Table 1). The three dimensional structures were evaluated for quality check which showed a Ramachandran plot value of 88.3–97.1% residues in allowed region. The Z-score and LG score were found to be between −4.6 to −7.47 and 3.879 to 5.396, respectively (Supplementary Table 1). Thus, the generated models were perfect and authentic for molecular docking. The molecular docking of the laccase isozymes with different substrates showed maximum interaction with ABTS (−74.26 to −98.57) followed by *O*-tolidine (−48.85 to −56.05), and guaiacol (−34.21 to −41.10) (Figure 9A). The *in-silico* approach used to demonstrate the binding affinity of laccase isozymes with assay substrates (ABTS) clearly indicates the maximum interaction, which validates our earlier work on few selected laccase isozymes (Glac H1, Glac L1) (Kumar et al., 2015) and present kinetics data on Glac L2, Glac L3, Glac L4, and Glac L5, produced from *G. lucidum* MDU-7. Furthermore, the interaction of the laccase substrates i.e., ABTS, *O*-tolidine, and guaiacol was found to be best with isozyme GL29486, GL29490, and GL29234, respectively (Figure 9B and Supplementary Figure 5). Binding affinity was also significantly higher and comparable to substrates binding with laccase models. Furthermore, the molecular docking and dynamics simulation tools have been used to study the differences in folding of laccase of *Populus trichocarpa* and *Trametes versicolor* to support their diversified role (Awasthi et al., 2015). Earlier, the *in-silico* approach has helped to understand the molecular structures of isozymes of heme peroxidases from whole genome of *Pleurotus ostreatus* (Ruiz-Dueñas et al., 2011).

### Effect of laccase on cotton callogenesis

**Figure 10:**
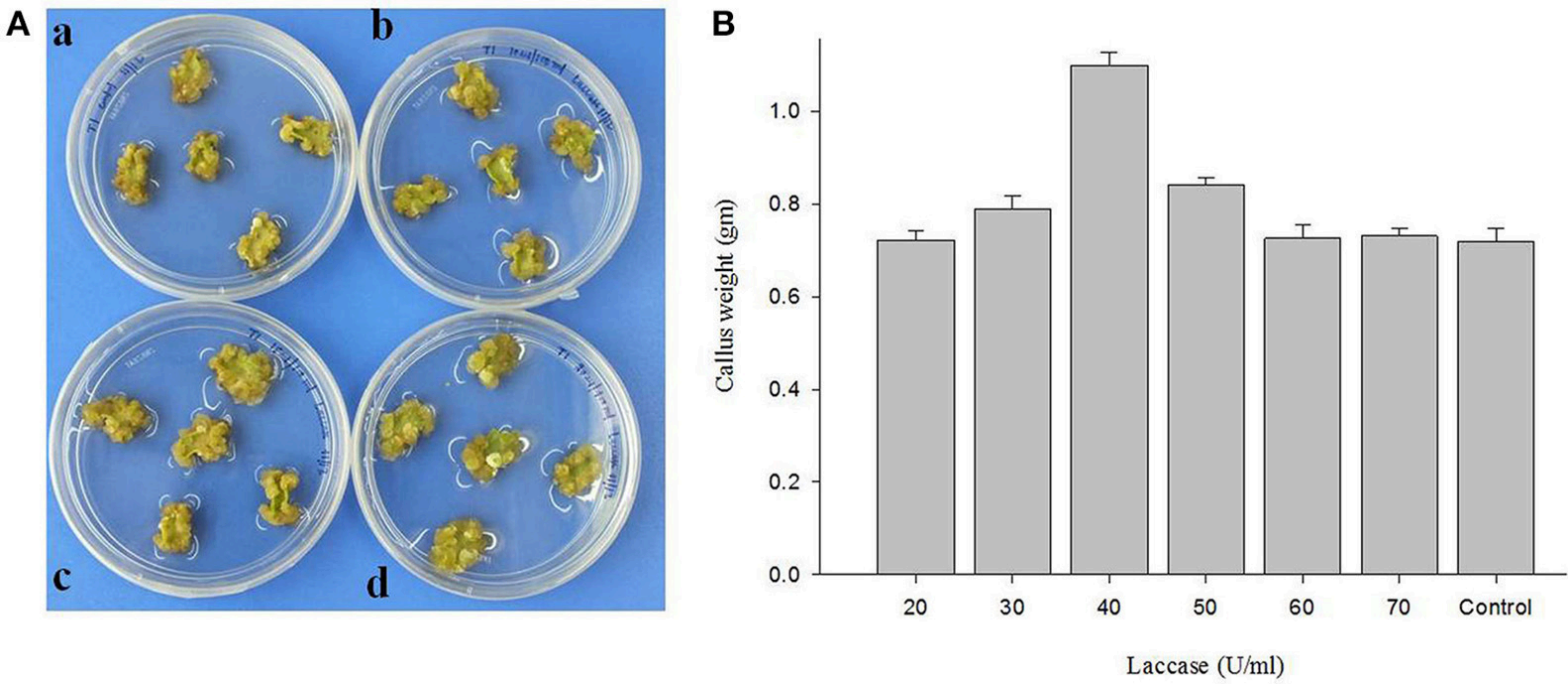
**The effect of laccase from ***G. lucidum*** MDU-7 on cotton callogenesis. (A)** Callogenesis from cotton cotyledonary tissues under different concentration of laccase in MST1 medium. (a), Control; (b), 20 U; ( c), 30 U; (d), 40 U of ml^−1^ of medium. **(B)** Evaluation of effect of various concentration of laccase on callus culture.

Different concentrations of copper induced laccase (20–70 U/ml of culture medium) having multiple isozymes was tested to assess the effect in callogenesis in cotton tissue (Figure 10B; Table 4). The results showed a variable rate of callus growth at different concentrations of laccase (Figure 10A). Significant callus induction was observed at 40 U/ml of laccase in the culture medium, with callus mass i.e., 1.09853 g ± 0.02806 than control i.e., 0.7178 g ± 0.03026 (Figure 10B).

**Table 4:** **Evaluation of effect of various concentration of laccase on callus culture**.

**Laccase concentrations (unit/ml)**	**Replicates**	**Degree of freedom**	**Mean callus weight (mg)**
20 U/ml	15	14	721.33 ± 21.42^a^
30 U/ml	15	14	787.60 ± 30.06^b^
40 U/ml	15	14	1098.53 ± 28.06^c^
50 U/ml	15	14	841.60 ± 15.07^d^
60 U/ml	15	14	725.07 ± 28.38^a^
70 U/ml	15	14	730.07 ± 16.90^a^
Control	15	14	717.80 ± 30.26^a^

*The data were shown in mean ± SD. values obtained for different concentration of laccase enzyme after carrying out one way ANOVA and further analyzing data using paired t-test analysis. P < 0.05 were considered as significantly different*.

a–d *Groups belonging to the same category denoted with same superscripts*.

**Figure 11:**
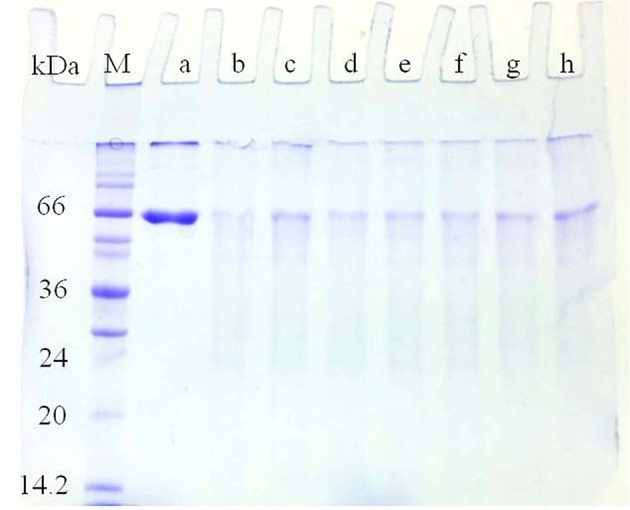
**Antioxidant properties of Glac H1-Glac L5 against BSA and Cu^**2+**^/H_**2**_O_**2**_ model system**. (a) 50 μg of BSA was added 0.1 ml of 100 μM copper and 2.5 mM H_2_O_2_; (b) 50 μg of ascorbate was added (a), 5 U of laccase isozymes i.e., (c) Glac H1, (d) Glac L1, (e) Glac L2, (f) Glac L3, (g) Glac L4, and (h) Glac L5 were added to (b), respectively.

Interestingly, the purified laccase isozymes showed antioxidative properties with BSA as a reference protein. Glac H1 and Glac L5 were found to be better the scavenger of free radicals (Figure 11). An increase in free radical due to many factors i.e., physical damage, saturation in antioxidant protection mechanism, stress, and pathological disease (Benson, 2000). Fungal laccase from *Polyporus versicolor* has been reported to form an adduct with the OH radical thus it behaves as a free radical scavenger (Guissani et al., 1982). Moreover, antioxidant i.e., glutathione, DL-α-tocopherol and sodium selenite have been reported to increase the explants number and transformation efficiency (Qiusheng et al., 2005). Differential behaviors of laccase isozymes are reported in different conditions i.e., virus infection, biotic stress, hormonal treatment or nutritional starving conditions (Turlapati et al., 2011). Interestingly, the plant laccases also show tissue specific expression in an early stage of root growth, leaf development, pollen grain development and in wound responses (Turlapati et al., 2011), overall, suggesting the multiple roles of laccase isozymes apart from lignification.

## Conclusion

Laccase isozymes from *G. lucidum* MDU-7 and *Ganoderma* sp. kk-02 studied under different cultural conditions showed a different enzyme yield and physicochemical properties. The structural studies of laccase gene family could be used to predict isozyme-specific catalytic role in the transformation of several phenolic and non-phenolic compounds. The MALDI-TOF peptide fingerprinting analysis of laccase isozymes reveals their multimeric nature. This information will aid in the knowledge about proteins with similar catalytic functions, molecular mass and differential surface charge. Furthermore, the biochemical properties of the laccase isozymes and specific induction time indicate the evolutionary compitentness of filamentous fungi and their colonizing success on diverse and complex phenolic and the non-phenolic substrates. The callogenesis of the plant tissue by fungal laccases further suggest the more reason for the fungal-plant association other than lignin degradation.

## Author contributions

Conceived and designed the experiments: KS, AK. Performed the experiments: AK, DS, SA, and AS. Analyzed the data: KS, AK, DS, AS, and BS. Wrote the paper: AK, DS, KS, AS, BS and SG.

### Conflict of interest statement

The authors declare that the research was conducted in the absence of any commercial or financial relationships that could be construed as a potential conflict of interest.

## Supplementary material

Supplementary Figure 1 **UV/Vis spectrum of the purified laccase isozymes from ***G. lucidum*** MDU-7**.

Supplementary Figure 2 **Michaelis-Menten constant (***K***_**m**_) and the maximum rate of reaction (V_**max**_) were calculated from Eadie-Hofstee plot for substrate ABTS**.

Supplementary Figure 3 **Michaelis-Menten constant (***K***_**m**_) and the maximum rate of reaction (V_**max**_) were calculated from Eadie-Hofstee plot for substrate guaiacol**.

Supplementary Figure 4 **Michaelis-Menten constant (***K***_**m**_) and the maximum rate of reaction (V_**max**_) were calculated from Eadie-Hofstee plot for substrates ***O***-tolidine**.

Supplementary Figure 5 **Molecular interaction of laccase isozymes with different substrates**. Interaction of (A) isozyme GL29486 with substrate ABTS; **(B)** isozyme GL29490 with substrate *O*-tolidine; **(C)** isozyme GL29234 with substrate guaiacol+.

Supplementary Table 1 **Quality check of laccase isozymes models using Modeller, ProSa, RAMPAGE, and ProQ**.
