# Supplementary figures and images for: Gel-Based Purification and Biochemical Study of Laccase Isozymes from Ganoderma sp. and Its Role in Enhanced Cotton Callogenesis
Source: Front Microbiol. 2017 Apr 20;8:674. doi: 10.3389/fmicb.2017.00674 (PMC5397484; doi:10.3389/fmicb.2017.00674)

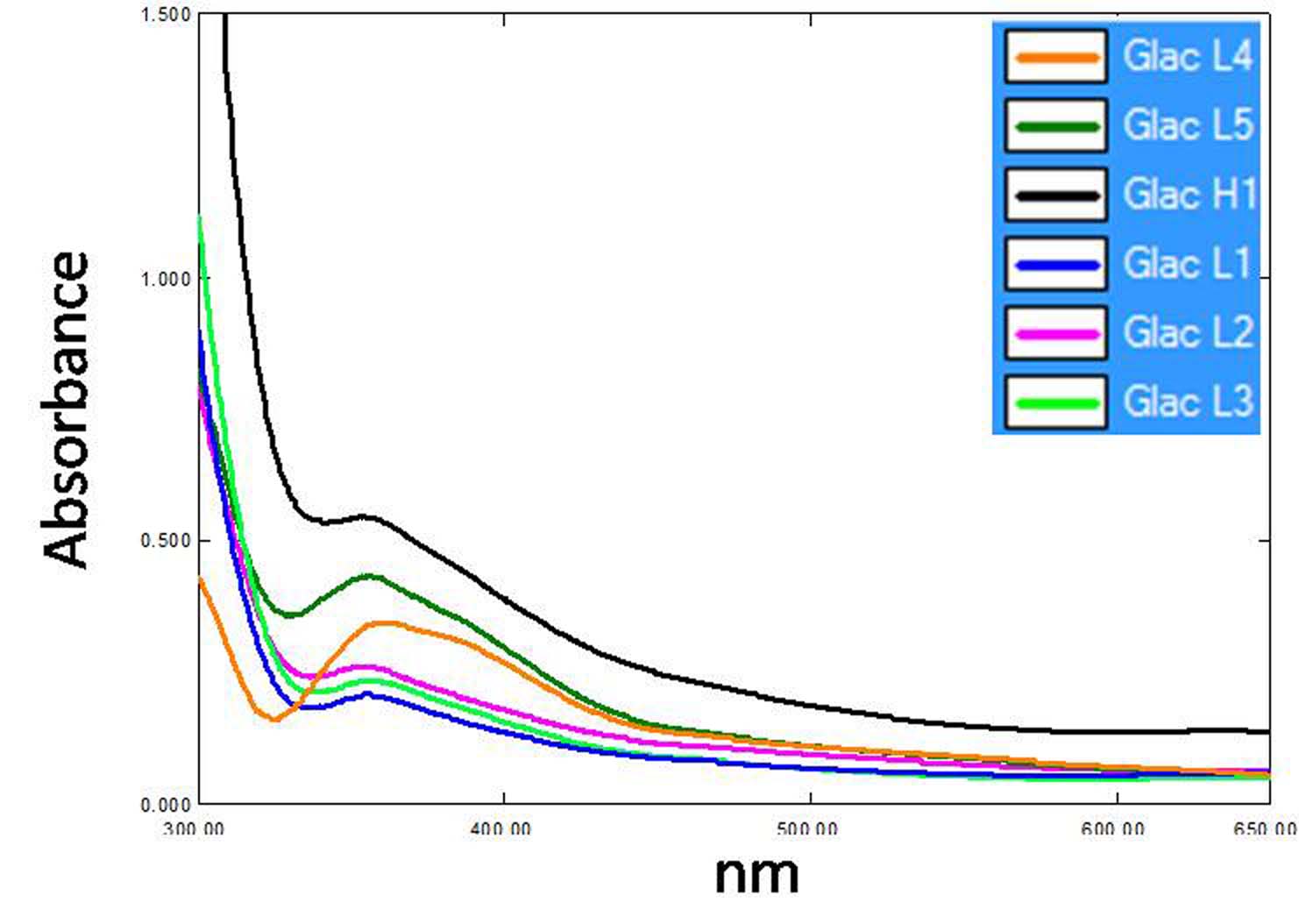

Supplement: Supplementary Figure 1 — UV/Vis spectrum of the purified laccase isozymes from G. lucidum MDU-7. [file Image1.JPEG]

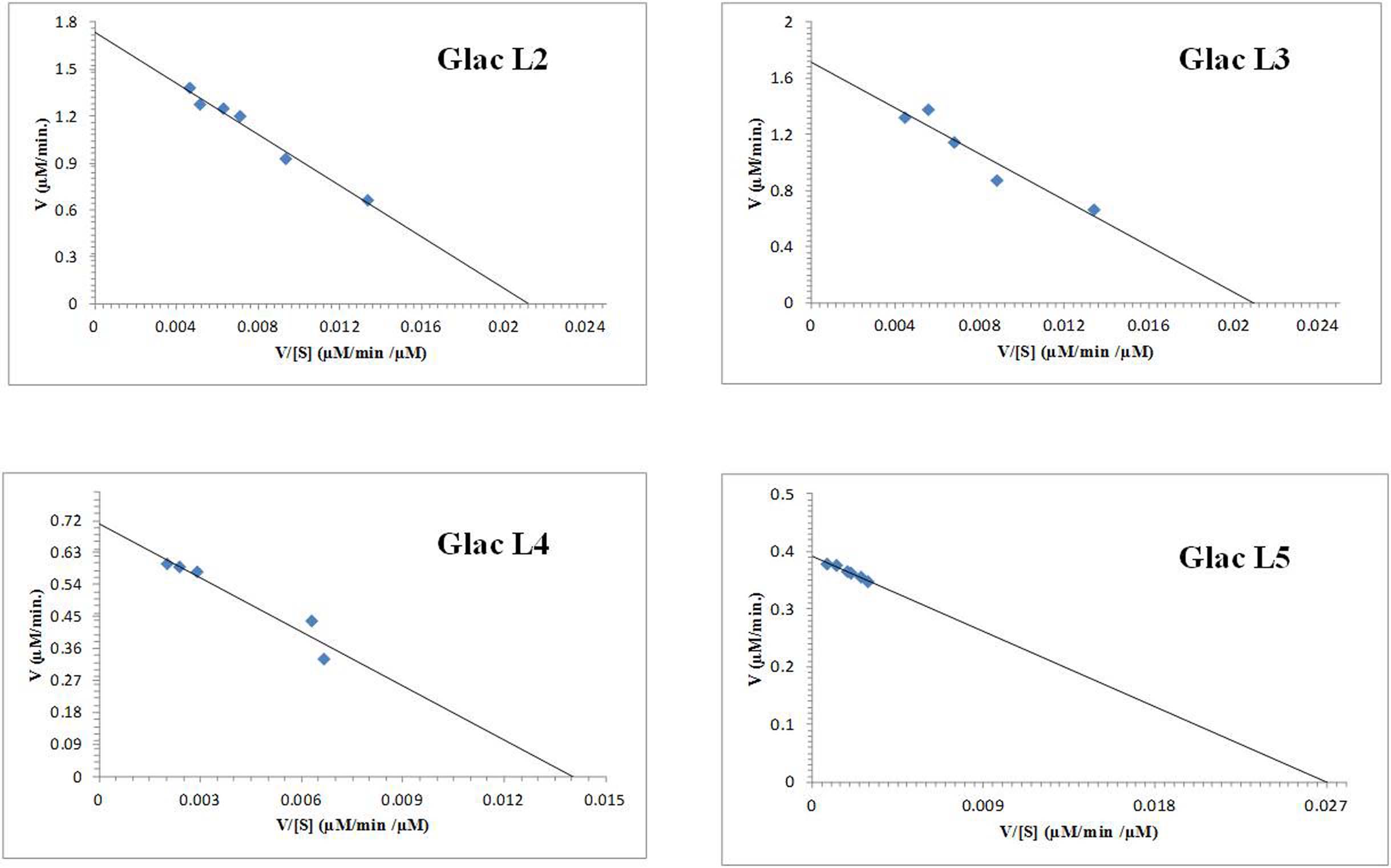

Supplement: Supplementary Figure 2 — Michaelis-Menten constant (Km) and the maximum rate of reaction (Vmax) were calculated from Eadie-Hofstee plot for substrate ABTS. [file Image2.JPEG]

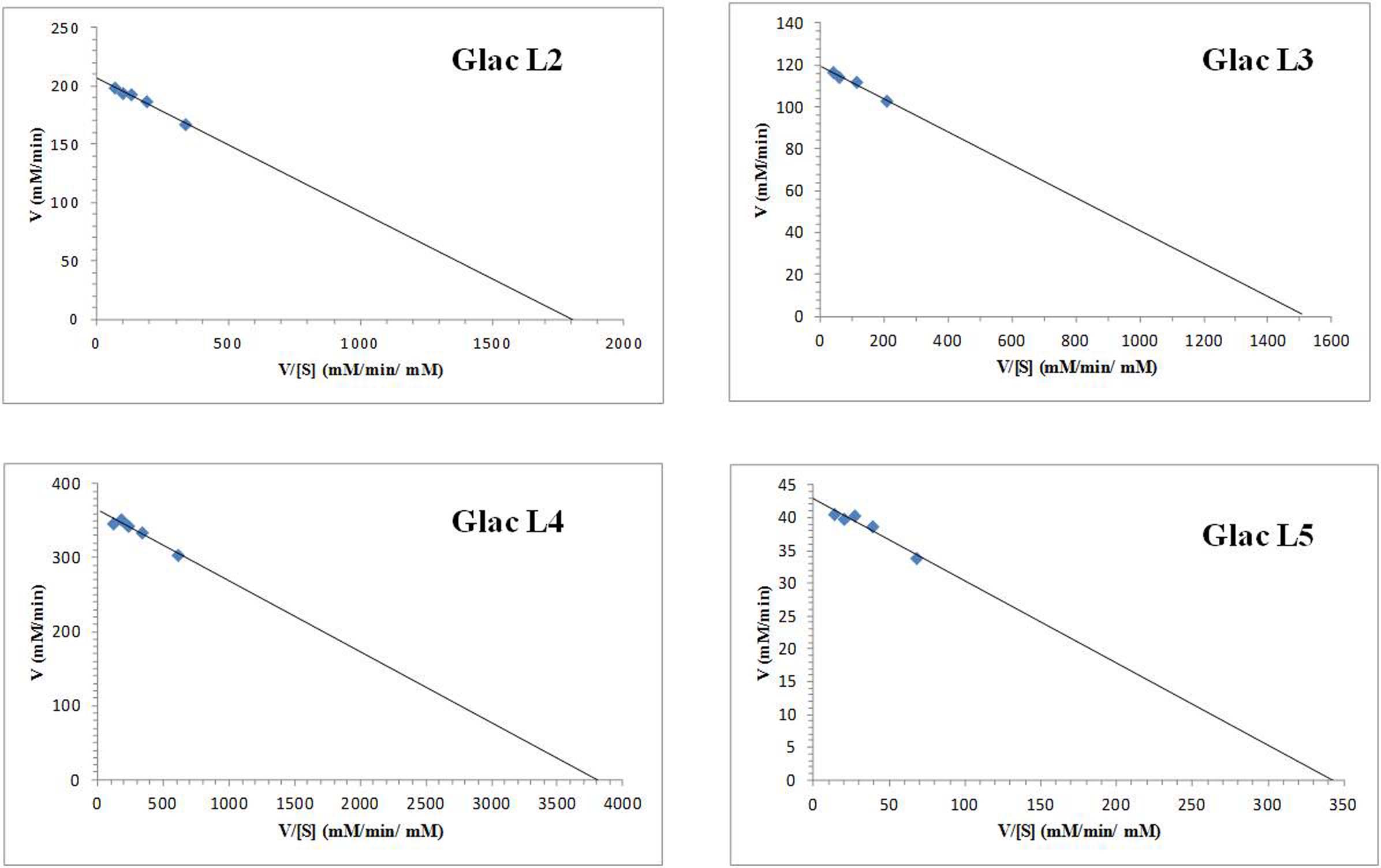

Supplement: Supplementary Figure 3 — Michaelis-Menten constant (Km) and the maximum rate of reaction (Vmax) were calculated from Eadie-Hofstee plot for substrate guaiacol. [file Image3.JPEG]

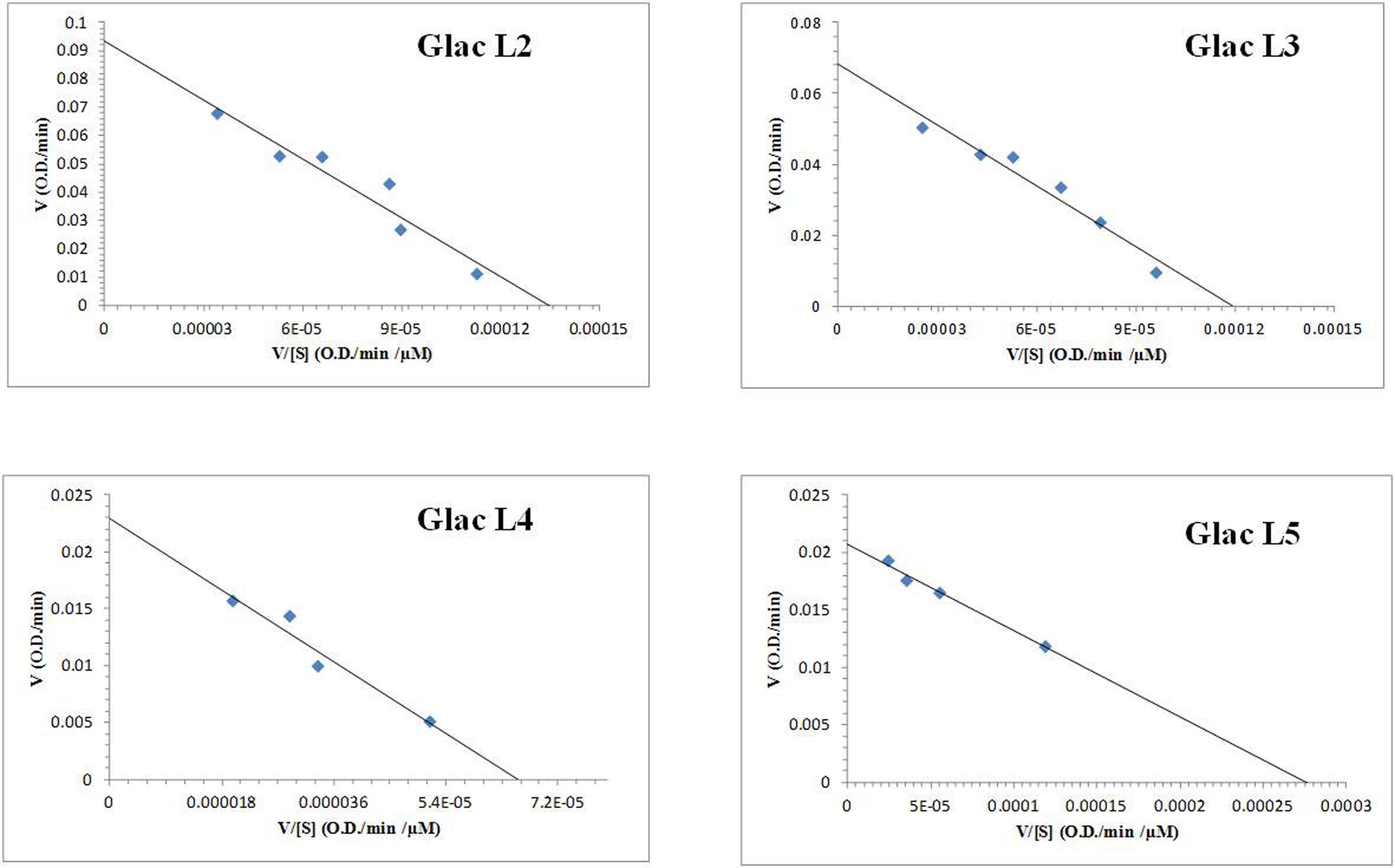

Supplement: Supplementary Figure 4 — Michaelis-Menten constant (Km) and the maximum rate of reaction (Vmax) were calculated from Eadie-Hofstee plot for substrates O-tolidine. [file Image4.JPEG]

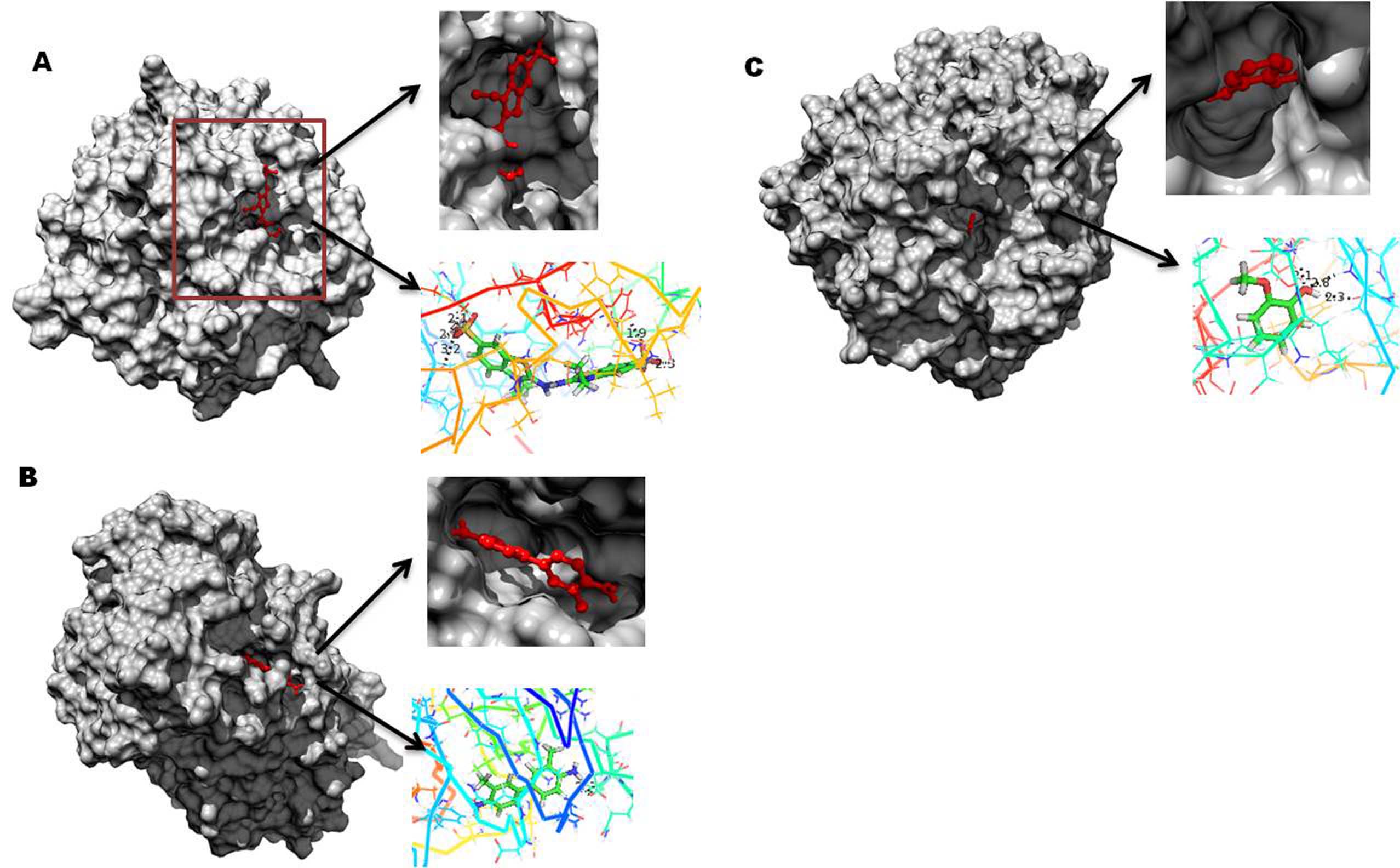

Supplement: Supplementary Figure 5 — Molecular interaction of laccase isozymes with different substrates. Interaction of (A) isozyme GL29486 with substrate ABTS; (B) isozyme GL29490 with substrate O-tolidine; (C) isozyme GL29234 with substrate guaiacol+. [file Image5.JPEG]
