# Supplementary material for: Gel-Based Purification and Biochemical Study of Laccase Isozymes from Ganoderma sp. and Its Role in Enhanced Cotton Callogenesis
Source: Front Microbiol. 2017 Apr 20;8:674. doi: 10.3389/fmicb.2017.00674 (PMC5397484; doi:10.3389/fmicb.2017.00674)
Supplement: Supplementary Table 1 — Quality check of laccase isozymes models using Modeller, ProSa, RAMPAGE, and ProQ. [file Table1.docx]

**Supplementary Table 1** Quality check of laccase isozymes models using Modeller, ProSa, RAMPAGE and ProQ

| **Isozyme** | **Mol pdf** | **DOPE Score** | **Z-Score** | **RP%** | **LG Score** |
| --- | --- | --- | --- | --- | --- |
| GL16398 | 3565.39502 | -62564.195312 | -6.49 | 95.9 | 5.005 |
| GL29486 | 3250.31226 | -62758.878906 | -7.47 | 97.1 | 5.123 |
| GL29253 | 2922.32227 | -61303.960938 | -6.73 | 96.9 | 5.028 |
| GL21497 | 3745.21826 | -64437.960938 | -5.91 | 93.9 | 4.826 |
| GL29490 | 3241.49854 | -59737.742188 | -7.4 | 96.6 | 4.960 |
| GL30788 | 4601.07666 | -59494.55.781 | -4.99 | 94.3 | 3.879 |
| GL29234 | 4129.99658 | -58838.421875 | -6.22 | 95.0 | 4.366 |
| GL23477 | 3579.82104 | -61669.203125 | -6.54 | 94.2 | 5.390 |
| GL17426 | 3396.59180 | -60695.363281 | -6.35 | 95.4 | 5.294 |
| GL18428 | 3480.88013 | -56397.367188 | -7.29 | 95.1 | 5.068 |
| GL16401 | 3784.60571 | -57737.917969 | -6.12 | 93.3 | 5.263 |
| GL22987 | 6531.14941 | -63706.582031 | -4.6 | 88.3 | 4.239 |
